# Danggui-Shaoyao-San (DSS) Ameliorates Cerebral Ischemia-Reperfusion Injury via Activating SIRT1 Signaling and Inhibiting NADPH Oxidases

**DOI:** 10.3389/fphar.2021.653795

**Published:** 2021-04-15

**Authors:** Yunxia Luo, Hansen Chen, Bun Tsoi, Qi Wang, Jiangang Shen

**Affiliations:** ^1^ Science and Technology Innovation Center, Guangzhou University of Chinese Medicine, Guangzhou, China; ^2^ School of Chinese Medicine, Li Ka Shing Faculty of Medicine, The University of Hong Kong, Hong Kong, China; ^3^ Department of Endocrinology, Fourth Clinical Medical College, Guangzhou University of Chinese Medicine, Shenzhen, China; ^4^ Institute of Clinical Pharmacology, Guangzhou University of Chinese Medicine, Guangzhou, China

**Keywords:** Danggui-Shaoyao-San, stroke, peroxynitrite, SIRT1, oxidative stress

## Abstract

Danggui-Shayao-San (DSS) is a famous Traditional Chinese Medicine formula that used for treating pain disorders and maintaining neurological health. Recent studies indicate that DSS has neuroprotective effects against ischemic brain damage but its underlining mechanisms remain unclear. Herein, we investigated the neuroprotective mechanisms of DSS for treating ischemic stroke. Adult male Sprague-Dawley (S.D.) rats were subjected to 2 h of middle cerebral artery occlusion (MCAO) plus 22 h of reperfusion. Both ethanol extract and aqueous extract of DSS (12 g/kg) were orally administrated into the rats at 30 min prior to MCAO ischemic onset. We found that 1) ethanol extract of DSS, instead of aqueous extract, reduced infarct sizes and improved neurological deficit scores in the post-ischemic stroke rats; 2) Ethanol extract of DSS down-regulated the expression of the cleaved-caspase 3 and Bax, up-regulated bcl-2 and attenuated apoptotic cell death in the ischemic brains; 3) Ethanol extract of DSS decreased the production of superoxide and peroxynitrite; 4) Ethanol extract of DSS significantly down-regulated the expression of p67^phox^ but has no effect on p47^phox^ and iNOS statistically. 5) Ethanol extract of DSS significantly up-regulated the expression of SIRT1 in the cortex and striatum of the post-ischemic brains; 6) Co-treatment of EX527, a SIRT1 inhibitor, abolished the DSS’s neuroprotective effects. Taken together, DSS could attenuate oxidative/nitrosative stress and inhibit neuronal apoptosis against cerebral ischemic-reperfusion injury via SIRT1-dependent manner.

## Introduction

Stroke is a major disease burden with high mortality and disability in which ischemic stroke accounts for 87% (Feigin et al., 2014; Feigin et al., 2015; Benjamin et al., 2017). To date, tissue plasminogen activator (t-PA) is the only United States Food and Drug Administration approved drug for acute ischemic stroke. With the narrow therapeutic window within 4.5 h and the risk of hemorrhagic transformation, less than 10% ischemic stroke patients benefit from t-PA treatment (Mozaffarian et al., 2016). Seeking new therapeutic approaches is timely important for ischemic stroke.

Reactive oxygen species (ROS) and reactive nitrogen species (RNS) are important players in cerebral ischemia-reperfusion injury. Superoxide $\left({\text{O}}_{2}^{-}\right)$ is representative ROS whereas nitric oxide (NO) and peroxynitrite (ONOO^−^) are typical RNS. During cerebral ischemia-reperfusion injury, the production of ${\text{O}}_{2}^{-}$ is mainly from the activations of NADPH oxidase (Miller et al., 2006), xanthine oxidase (McCord, 1985), and cyclooxygenase (COX) (Fabian et al., 1995; Kawano et al., 2006). NO is generated by the activation of endothelial nitric oxide synthase (eNOS), neuronal NOS (nNOS) inducible NOS (iNOS). The simultaneous presentation of NO and ${\text{O}}_{2}^{-}$ rapidly produces ONOO^−^ in a diffusion-limited rate (Chen et al., 2013). Peroxynitrite could mediate neural apoptotic cell death, and aggravate the blood-brain barrier (BBB) disruption, infarction enlargement and neurological deficit in cerebral ischemia-reperfusion injury (Chen et al., 2013). Peroxynitrite induces protein tyrosine nitration by the addition of a nitro group to the hydroxyl group of the tyrosine residue to form 3-nitrotyrosine (3-NT), a footprint marker for ONOO^−^ production (Kuhn et al., 2004). Plasma 3-NT level was positively correlated with the magnitude of the brain injury in ischemic stroke patients (Bas et al., 2012). ONOO^−^ could be a promising therapeutic target to attenuating neural cell death, protecting the BBB integrity, and reducing thrombolysis-mediated hemorrhage transformation for improving ischemic stroke outcome (Gong et al., 2015; Chen et al., 2018; Chen et al., 2020). Peroxynitrite decomposition catalysts reduce 3-NT expression and MMPs activation, attenuate hemorrhagic transformation and improve neurological outcome in ischemic rat brains with delayed t-PA treatment (Chen et al., 2015). Therefore, antioxidant therapy could be a promising therapeutic strategy for ischemic stroke treatment.

Silent information regulator 2 homolog 1 (SIRT1) plays crucial roles in the molecular regulations under oxidative/nitrosative stress related brain damages. SIRT1 is a protein deacetylase to regulating endothelium-dependent relaxation of the cerebral vasculature (Tajbakhsh and Sokoya, 2012). SIRT1 could be a therapeutic target in vascular-related diseases for restoring endothelial function. Under bilateral common carotid artery stenosis (∼50% stenosis), overexpression of SIRT1 preserves cerebral blood flow (CBF) via the deacetylation of eNOS (Hattori et al., 2014; Hattori et al., 2015). In bilateral common carotid artery occlusion (BCAO) mouse model, sirt1-overexpression significantly lessens ischemic brain damage with the preserved CBF up to 45–50% of the baseline level (Hattori et al., 2015). In a rat model of right-sided endovascular middle cerebral artery occlusion, activating SIRT1 decreased the infarct volume by targeting p53/microRNA-22 signaling pathway (Lu and Wang, 2017). Many antioxidants activate SIRT1 signaling for their neuroprotective effects (Wang et al., 2009; He et al., 2017; Ren et al., 2019; Teertam et al., 2020). Therefore, SIRT1 could be a promising therapeutic target for ischemic stroke.

Traditional Chinese Medicine (TCM) practice provides valuable sources for stroke treatment with relatively low- or non-toxicity (Wu et al., 2007; Seto et al., 2016). Danggui-Shaoyao-San (DSS), also called Tokishakuyaku-san (TJ-23) or Dangguijakyak-san (DJS), is a classic herbal formula including *Angelica sinensis (Oliv.) Diels (Umbelliferae), Paeonia lactiflora Pall. (Paeoniaceae), Conioselinum anthriscoides “Chuanxiong” (syn. Ligusticum chuanxiong. Hort.) (Umbelliferae), Wolfiporia extensa (Peck) Ginns (syn. Poria cocos (Schwein.) (Polyporaceae), Atractylodes. macrocephala Koidz. (Asteraceae)*, and *Alisma plantago-aquatica subsp. orientale (Sam.) Sam. (syn. Alisma orientalis (Sam.) Juzep.) (Alismataceae)* which forms a TCM formula mixed in a ratio of 3:16:8:4:4:8. DSS was originally used for gynecological diseases (Wang et al., 2015; Lee et al., 2016). Previous studies indicate the potentials of DSS for improving neurological functions in post stroke treatment (Goto et al., 2011; REN et al., 2013). DSS exerts various neuroprotective effects by ameliorating oxidative stress in a permanent ischemic stroke rat model and reducing inflammation in a global ischemia-reperfusion model (Lin et al., 2008; Kim et al., 2016). DSS treatment also promotes focal angiogenesis and neurogenesis, attenuates neurological deficit scores, and improves memory functions in experimental rat models of cerebral ischemic reperfusion injury (Izzettin et al., 2007; Song et al., 2013; Ren et al., 2015). However, the underlying mechanisms of DSS for neuroprotection remain largely unknown. In the present study, we tested the hypothesis that DSS could protect against cerebral ischemic-reperfusion injury via attenuating oxidative/nitrosative stress and inhibiting neuronal apoptosis in a SIRT1-dependent manner.

## Materials and Methods

### DSS Extraction Preparation

*Herbal materials including Angelica sinensis (Oliv.) Diels (Umbelliferae), Paeonia. lactiflora Pall. (Paeoniaceae), Conioselinum anthriscoides “Chuanxiong” (syn. Ligusticum chuanxiong Hort.) (Umbelliferae), Wolfiporia . extensa (Peck) Ginns (syn. Poria cocos (Schwein.) (Polyporaceae), Atractylodes macrocephala Koidz. (Asteraceae),.* and *Alisma plantago-aquatica subsp. orientale (Sam.) Sam. (syn. Alisma orientalis (Sam.) Juzep. (Alismataceae) were purchased from native sources* from Mainland China through School of Chinese Medicine, The University of Hong Kong, and these herbs were mixed in a ratio of 3:16:4:8:4:8 for extract preparation. We prepared both aqueous and ethanol extract to compare their effects in treating ischemic brain injury. The aqueous extract of DSS was prepared with the following procedure. The DSS was soaked in eight times of distilled water for 40 min following by decocted 1 h. After that, the filtrate was collected, and the filter residue was decocted with six volumes of distilled water for another 1 h. The filtrate was collected again and the two filtrates were mixed, lyophilized, and stored for usage. Ethanol extract of DSS preparation was made with the following procedures: Raw materials of DSS were ground into powder, macerated overnight and repeatedly ultrasound-extracted with 70% ethanol/water (1:10 w/v, 1:8 w/v, 1:5 w/v, respectively) for 1 h each time. The extracted solutions were evaporated under vacuum (45 °C) to remove ethanol, and the remained aqueous solution was frozen and freeze-dried to obtain DSS ethanol extract powder.

### Quality Control Analysis for DSS Ethanol Extract

Ethanol extract of DSS was analyzed by using high-performance liquid chromatography system **(**HPLC) in which paeoniflorin, alibiflorin, and ferulic acid were used as quantitative stands. Briefly, DSS power (200 mg) was accurately weighed, dissolved in 2 ml methanol proceed by sonication for 20 min and filtrated with 0.22 μm filter for quantitative analysis. DSS solution (5 μl) was injected into an apparatus with an autosampler. Chromatographic separation was achieved at a flow rate of 1.0 ml/min with an Agilent Eclipse Plus C18 column (4.6 × 250 mm, 5 µm). The details of mobile phase are shown in Supplementary Table S1. The separation temperature was 25°C, with a detection wavelength of 230 nm.

We detected the linearity, sensitivity, precision, accuracy, and stability for the validation of the quantitative methodology (Li et al., 2018) with a mini modification. In briefly, stock solutions of paeoniflorin (5,000 μg/ml), alibiflorin (620 μg/ml) and ferulic acid (180 μg/ml) were prepared in methanol. To prepare calibration curves, we analyzed seven concentrations of paeoniflorin, alibiflorin, and ferulic acid standers by using HPLC. The accuracy and precision were evaluated by measuring the intraday variabilities and recovery of those standard compounds. Stability was examined by analyzing DSS over a period of 0, 3, 6, 9, 12, and 24 h. The limits of detection (LOD) and limits of quantitation (LOQ) under the present conditions were determined at an S/N (signal/noise) of about 3 and 10, respectively. The data were monitored, recorded and analyzed by Agilent 1260 (United States).

### Cerebral Ischemia Reperfusion Injury Model

Adult male Sprague-Dawley (S.D.) rats (270–290 g) were obtained from the Laboratory Animal Unit, the University of Hong Kong. All procedures for animal care and experimental were approved by the University Committee on the Use of Live Animals in Teaching and Research (CULATR). The rats were kept in a temperature and humidity-controlled environment for 12 h dark/light cycles with free access to food and water.

Rats were subjected to middle cerebral artery occlusion (MCAO) to induce experimental cerebral ischemia-reperfusion model with the protocols as described previously with minor modification (Chen et al., 2015). Briefly, rats were anesthetized firstly with 4% isofluorane and maintained at 2% isofluorane through inhalation. A middle incision was made in the neck, followed by careful exposure of the left common carotid artery (CCA), external carotid artery (ECA), and internal carotid artery (ICA) under the microscope. A silicon-coated suture (Doccol, Redlands, CA, United States), with the diameter is 0.38 mm, was inserted from ECA to ICA, and advanced to occlude the middle cerebral artery (MCA). After 2 h of occlusion, the suture was removed and CCA was released to allow reperfusion. Sham group rats underwent the same surgical procedure without MCA occlusion. Rats body temperature were monitored during and after surgery. Rats were temporarily transferred to a cage with a heating lamp from recovery. 2,3,5-triphenyl-2H-tetrazolium chloride (TTC) staining was performed to evaluate the success of the MCAO model (Chen et al., 2020).

### Experimental Design and Drug Treatment

We investigated the neuroprotective effects of DSS ethanol extract (DSS/E) and aqueous extract (DSS/W) against cerebral ischemia-reperfusion injury. Rats were randomly divided into the following four groups: Sham control, MCAO, and MCAO plus DSS/W (12 g/kg wt), MCAO + DSS/E (12 g/kg wt). The dosage of 12 g/kg was equivalent to human doses of raw materials (Zhang, 2005). DSS/W or DSS/E (12 g/kg) was orally administered to the rats at 30 min before reperfusion. For sham and MCAO vehicle groups, rats were orally given the same volume of double-distilled water. Secondly, in order to elucidate whether the neuroprotective effects of DSS/E were SIRT1-dependent, rats were randomly divided into the following three groups: MCAO, MCAO plus DSS/E, MCAO plus EX527 and DSS/E. The rats in the MCAO vehicle and MCAO plus DSS groups were given the same treatment as described in the first experiment. For MCAO + EX527 + DSS group, the rats were intraperitoneally injected with EX527 at the dose of 5 mg/kg every 2 days for four times before MCAO surgical procedure (Kou et al., 2017).

### Neurological Deficit Cores

We used the modified Neurological Severity Score (mNSS) method to measure neurological deficits. The mNSS score was graded from 0 to 18, representing various levels of neurological dysfunction involving motor, sensory and reflex (Chen et al., 2001). The higher the score, the more severe neurological deficits. An investigator blind to the experimental design performed the mNSS test.

### Infarct Size Measurement

We evaluated cerebral infarct size by using 2,3,5-triphenyl-2H-tetrazolium chloride (TTC) method (Feng et al., 2018). Rats were anesthetized and perfused with PBS and then brain tissue harvest. Tissue sample was cut into 2-mm thick coronal slices, which were immediately immersed into 0.5% TTC (T8877, Sigma) solution at room temperature in the dark for 20 min. Digital images of the brain slices were captured using a camera, and the infarct size was measured and analyzed by using Image J software. To reduce the bias of brain edema, we calculated the infarct size with the following formula: Infarct size percentage = (right hemisphere − red size of left hemisphere)/right hemisphere size × 100%.

### TUNEL Staining

Apoptotic cell death was determined by using terminal deoxynucleotidyl transferase-mediated dUTP nick end labeling (TUNEL) assay. Briefly, rat brain samples were fixed with 4% paraformaldehyde (PFA) and then immersed in 30% sucrose until it sank. Samples were then embedded in O.C.T. and cut into a section of 25 μm. TUNEL staining was conducted referring to the manufacturer’s instructions in the TUNEL assay kit (Shanghai YEASEN Biotechnology Co.). Hoechst staining was used to visualize the cell nucleus. A fluorescence microscope (Carl Zeiss) with Axio Vision digital imaging system was applied to obtain the fluorescence images.

### Immunostaining

Immunostaining assay was performed to visualize the expressions of SIRT1, 3-nitrotyrosine (3-NT), and cleaved caspase-3. Brain samples were prepared as described in “*TUNEL Staining*” section. Samples were blocked with 5% goat serum (Thermo Fisher Scientific) in PBS and incubated with the primary antibodies including SIRT1 (1:200, Abcam), 3-NT (1:100, Abcam), and cleaved caspase-3 (1:100, Immunoway), at appropriate dilution overnight at 4°C. Then sections were incubated with secondary antibody Alexa Fluor 568 Goat anti-mouse (Invitrogen), Alexa Flor 488 Goat anti-rabbit, and Alexa Flor 647 Goat anti-mouse at room temperature for 2 h. DAPI ((4′,6-diamidino-2-phenylindole) was used for cell nucleus visualization. Immunofluorescent figures were obtained by a confocal microscope Carl Zeiss LSM 780.

### Western Blot Analysis

Western blot analysis was performed according to standard protocol. Briefly, brain tissues were lyzed in RIPA buffer containing 1% protease and phosphorylate inhibitor cocktail (Sigma-Aldrich). To determine protein concentration, an equal amount of total protein was separated by 10% sodium dodecyl sulfatepolyacrylamide (SDS-PAGE) gel electrophoresis and transferred to polyvinylidene fluoride membranes (IPVH00010, EMD Millipore, Germany). Membranes were blocked with 5% bovine serum albumin and then probed with a primary antibodies including β-actin (Mouse, 1:3,000, Sigma), iNOS (Rabbit, 1:200, Abcam), nNOS (Rabbit, 1:1,000, Abcam), Cleaved-caspase3 (Rabbit polyclonal, 1:1,000, Millipore), caspase3 (Rabbit, 1:500, Abcam) or 3-NT (Mouse, 1:1,000, Millipore) overnight at 4°C. The membranes were washed by using TBS-Tween 20 buffer and incubated with the secondary antibody (1:2,000) for 2 h at room temperature. The immunoblots were enhanced using chemiluminescent ECL select kit (GE Healthcare, IL, United States), detected by Gel-Doc system (Bio-Rad, CA, United States) and analyzed with Image Lab software (Bio-Rad, CA, United States).

### Superoxide Detection

We detected the superoxide production by using hydroethidine (HEt) and HKSOX-1, a newly developed high specific and sensitive fluorescent probe (Hu et al., 2015). The isolated brains were immediately made into frozen sections, and the brain slice at 6 mm from the frontal tip was stained with the probe solutions of HEt (20 μM,DMF) or HKSOX-1 (20 μM,DMF) for 10 min in the dark. Fluorescence was immediately detected by using Carl Zeiss LSM 780 Confocal Microscopy.

### Statistical Analysis

Data were represented as Mean ± SEM. Statistical analysis was performed by using one-way analysis of variance (ANOVA) followed by Dunnett’s multiple-comparison test. Neurological severity scores were analyzed by using non-parametric Kruskal-Wallis tests, followed by Dunnett’s multiple comparison test. *p* < 0.05 was considered as statistically significant.

## Results

### Ethanol Extract of DSS had Better Neuroprotective Effects than Aqueous Extract in Cerebral Ischemia-Reperfusion Injury

**FIGURE 1:**
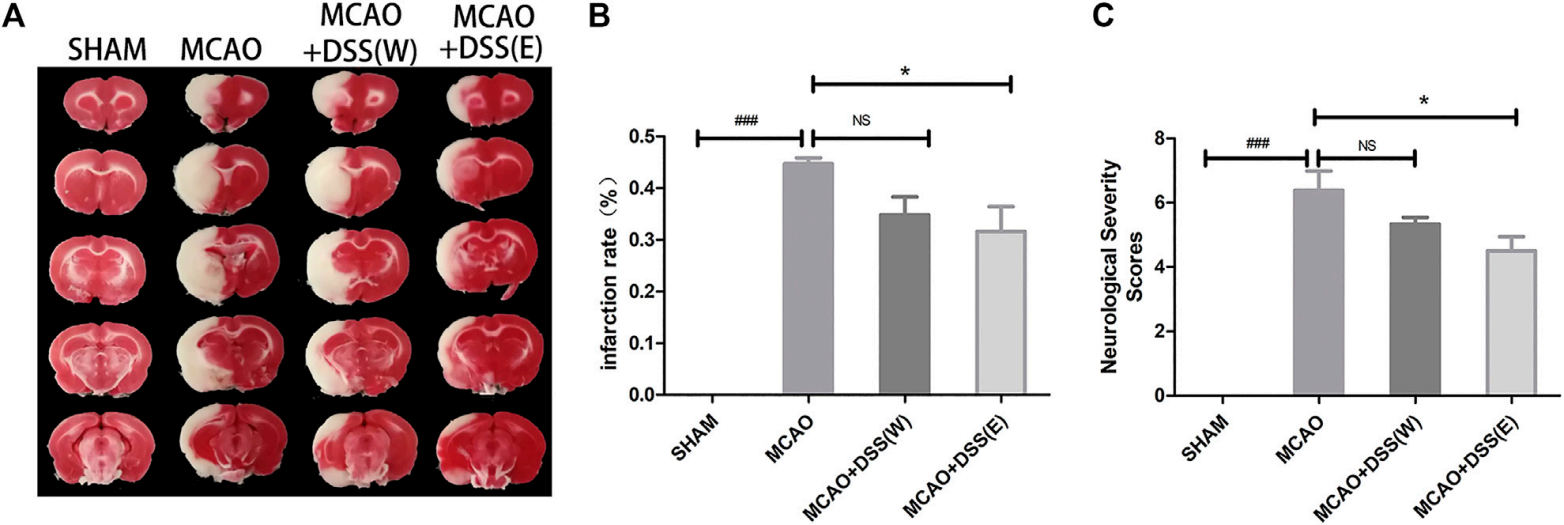
Effects of DSS ethanol extract [DSS(E)] and aqueous extract [DSS(W)] on infarction sizes and neurological deficit scores in post MCAO ischemic rats. S.D. rats were allocated into groups of sham control, MCAO, and MCAO + DSS (W) and MCAO + DSS (E). The rats for MCAO or MCAO plus DSS treatment group were subjected to 2 h of MCAO ischemia plus 22 h of reperfusion. Aqueous and ethanol extracts of DSS at the dosage of 12 g/kg was orally administrated into the rats at 30 min before reperfusion after 1.5 h MCAO ischemia. Sham: sham control group; MCAO: the rat group was subjected to 2 h of MCAO ischemia plus 24 h of reperfusion; MCAO + DSS (E): MCAO plus DSS ethanol extract treatment (12 g/kg). MCAO + DSS (W): MCAO plus DSS aqueous extract treatment (12 g/kg). Infarct sizes and neurological deficit scores were evaluated. **(A)** Representative brain slices of TTC staining for infarct sizes. The white color area represents brain infarction area. **(B)** Statistical analysis of infarct sizes as measured by using ImageJ software, *n* = 8–9, vs. MCAO, *p < 0.05. **(C)** Neurological deficit was measured by using the modified Neurological Severity Scores (mNSS) in each group at 24 h after MCAO ischemia, *n* = 8–9, vs. MCAO *p < 0.05.

We firstly compared the neuroprotective effects of DSS with ethanol extract [DSS(E)] and aqueous extract [DSS(W)]. Rats were subjected to 2 h MCAO ischemia plus 22 h reperfusion. We analyzed infarct size and examined neurological deficit scores in the MCAO ischemia-reperfused rats with or without DSS treatment. As shown in Figure 1, DSS(E) treatment significantly reduced the infarct sizes and neurological deficit mNSS scores whereas DSS(W) treatment had no neuroprotective effects. Therefore, the ethanol extract of DSS, instead of aqueous extract, has neuroprotective effects against cerebral ischemic-reperfusion injury.

### DSS Ethanol Extract Inhibited Cleaved-Caspase3 and Bax, and Attenuated Apoptotic Cell Death in Ischemia-Reperfused Rat Brains

**FIGURE 2:**
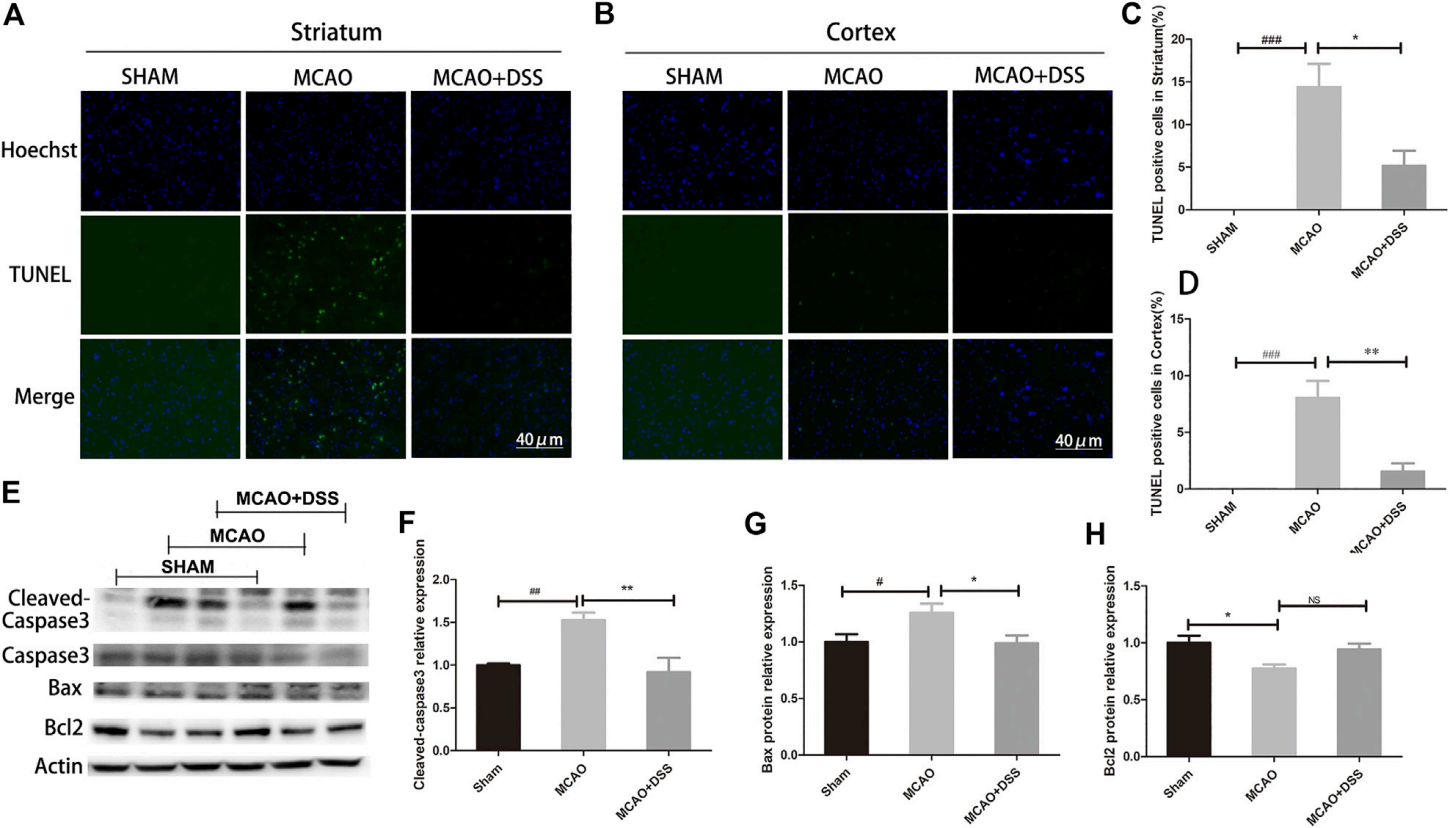
**|** DSS ethanol extract inhibited the expression of the cleaved-caspase 3 and Bax, and attenuated apoptotic cell death in ischemia-reperfused rat brains. DSS ethanol extract was simply named as DSS. Sham: sham control group; MCAO: MCAO ischemia-reperfusion group; MCAO + DSS: MCAO plus DSS ethanol extract treatment. Th rats were subjected to 2 h of MCAO ischemia plus 22 h of reperfusion. The DSS ethanol extract at the dosage of 12 g/kg was orally administrated into the rats at 30 min before reperfusion after 1.5 h MCAO ischemia. **(A)** Co-immunostaining TUNEL (green) and DAPI (blue) in the striatum of the ipsilateral side. **(B)** Co-immunostaining TUNEL (green) and DAPI (blue) in the cortex of the ipsilateral side. **(C)** Statistic analysis of TUNEL positive cells in the striatum of the ipsilateral side. Versus Sham control, ^###^ *p* < 0.001; Versus MCAO, *p < 0.05, *n* = 4. **(D)** Statistic analysis of TUNEL positive cells in the cortex of the ipsilateral side. Versus Sham control, ^###^ *p* < 0.001; Versus MCAO, **p < 0.01, *n* = 4. **(E)** Representative immunoblot results of cleaved-caspase 3, Bax and Bcl-2 in the brain tissues. **(F)** Statistic analysis of cleaved-caspase3 expression in the brain tissues. Versus Sham control, *p < 0.05, *n* = 8. **(G)** Statistic analysis of Bax expression in the brain tissues. Versus Sham control, ^#^ *p* < 0.05; Versus MCAO, *p < 0.05, *n* = 8. **(H)** Statistic analysis of Bcl-2 expression in the brain tissues, *n* = 8.

We then investigated the effects of the DSS ethanol extract on apoptotic cell death in acute MCAO ischemia reperfused brains. We used the DSS ethanol extract for the rest of the experiments whose name was simplified as DSS accordingly. TUNEL staining was used to evaluate apoptotic cell death in the ischemic brain tissues at 22 h after 2 h of MCAO ischemia. As shown in Figure 2, DSS treatment significantly decreased apoptotic cell death in both cortex and striatum of the ischemia-reperfusion brains. In line with the result of TUNEL staining, western blot analysis showed that DSS down-regulated the expression of the cleaved-caspase 3 and Bax but up-regulated the expression of bcl-2 in the ischemic brains. These results suggest that DSS ethanol extract inhibits apoptotic cell death in cerebral ischemia-reperfusion injury.

### DSS Ethanol Extract Decreased Superoxide Level and Inhibited 3-Nitrotyrosine Expression in Ischemia-Reperfused Rat Brains

**FIGURE 3:**
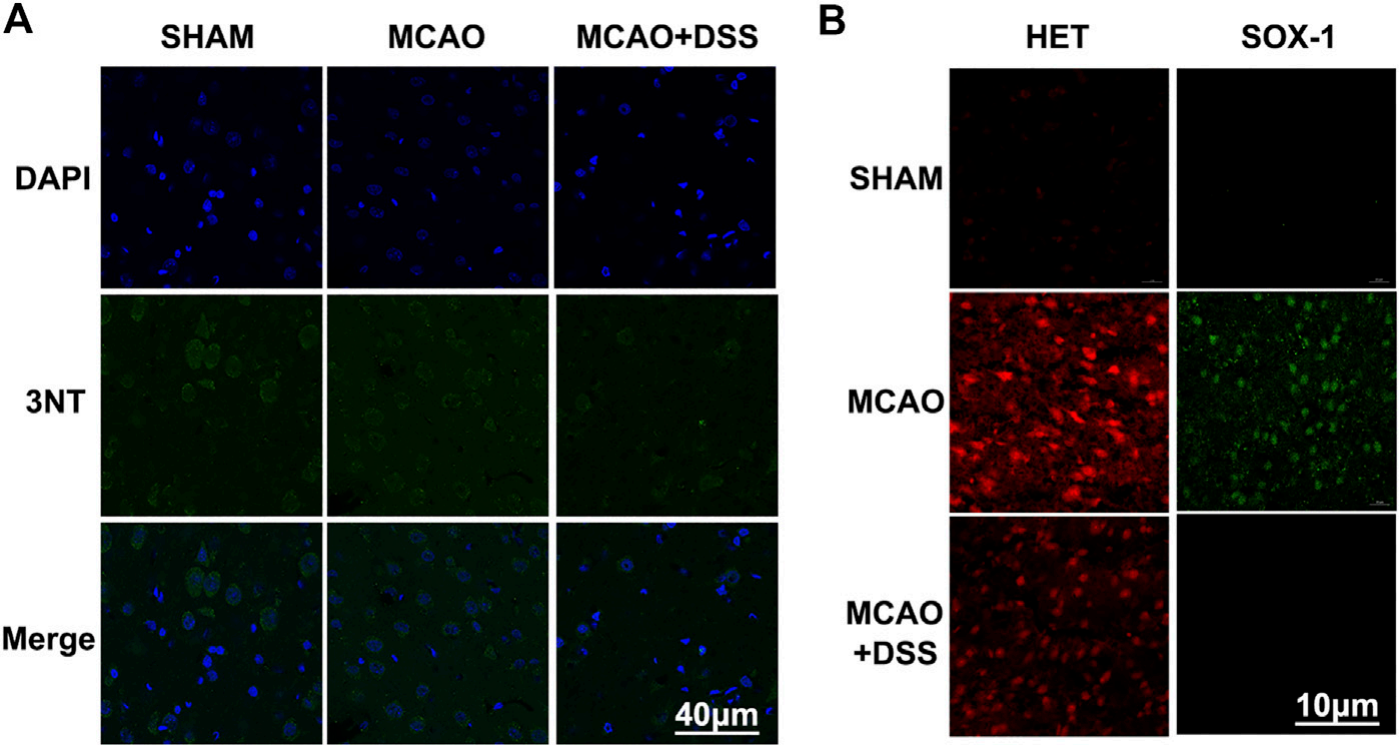
DSS ethanol extract decreased superoxide and peroxynitrite in ischemic-reperfused rat brains. DSS ethanol extract was simply named as DSS. Sham: sham control group; MCAO: MCAO ischemia-reperfusion group; MCAO + DSS: MCAO plus DSS ethanol extract treatment. Th rats were subjected to 2 h of MCAO ischemia plus 22 h of reperfusion. The DSS ethanol extract at the dosage of 12 g/kg was orally administrated into the rats at 30 min before reperfusion after 1.5 h MCAO ischemia. (**A)** Representative immunofluorescent imaging of 3-nitrotyrosine (3-NT) (green), *n* = 4. (**B)** Representative immunofluorescent imaging of HKSOX-1 (green) and HEt (red) for detecting superoxide, *n* = 4.

We then investigated the antioxidant properties of DSS to scavenging ${\text{O}}_{2}^{-}$ and ONOO^−^ in the rat brains after subjected to 2 h MCAO ischemia plus 22 h reperfusion. The production of ${\text{O}}_{2}^{-}$ were detected by using HE_t_ and HKSOX-1 (Hu et al., 2015). The production of ONOO^−^ was examined by the immunostaining of 3-NT, a footprint protein of ONOO^−^. As shown in Figure 3, the DSS treatment group had a significantly lower expression level of 3-NT and lower fluorescent staining of HE_t_ and HKSOX-1 in the ischemic brains than the MCAO vehicle treatment group. Those results suggest that DSS could inhibit the productions of superoxide and peroxynitrite in cerebral ischemia-reperfusion injury.

### DSS Ethanol Extract Inhibited NADPH Oxidase and Up-Regulated SIRT1 Expression in Ischemic-Reperfusion Rat Brains

**FIGURE 4:**
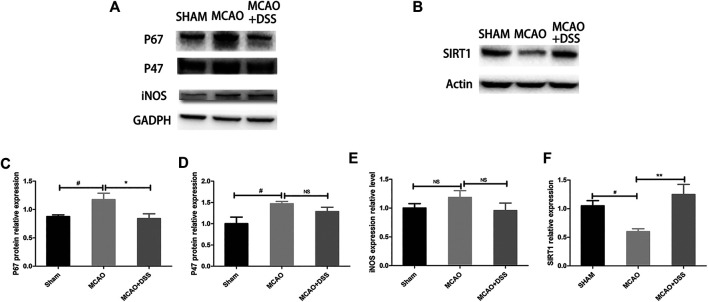
DSS treatment inhibited the expression of NADPH oxidase subunit p67^phox^ and up-regulated SIRT1 but had no effect on p47^phox^ and iNOS in MCAO ischemic brains. Sham: sham control group; MCAO: MCAO ischemia-reperfusion group; MCAO + DSS: MCAO plus DSS ethanol extract treatment. The rats were subjected to 2 h of MCAO ischemia plus 22 h of reperfusion. The DSS ethanol extract at the dosage of 12 g/kg was orally administrated into the rats at 30 min before reperfusion after 1.5 h MCAO ischemia. **(A)** Representative immunoblot results of p67^phox^, p47^phox^, and iNOS; **(B)** Representative immunoblot results of SIRT1; **(C–F)** Statistical analysis for the expressions of p67^phox^, p47^phox^, iNOS and SIRT1. Vs. Sham control, #*p* < 0.05, Vs. MCAO, *p < 0.5, **p < 0.01, *n* = 4 in each group.

**FIGURE 5:**
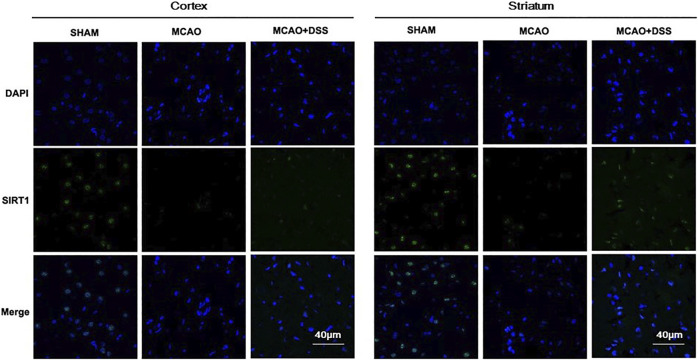
DSS up-regulated the expression of SIRT1 in cortex and striatum of the ischemia-reperfused brains. Representative immune staining (green) for SIRT1 in cortex (left) and striatum (right) of the ipsilateral side in each group; blue represents DAPI positive signal.

NADPH oxidase and iNOS are major enzymes for the productions of superoxide and nitric oxide respectively in cerebral ischemia-reperfusion injury (Robinson et al., 2011; Winterbourn et al., 2016). Meanwhile, SIRT1 exerts neuroprotective effects by attenuating oxidative stress in ischemic brain injury (Shin et al., 2012; Fu et al., 2014). SIRT1 could be also a promising therapeutic target for ischemic stroke (He et al., 2017; Lu and Wang, 2017; Ren et al., 2019; Teertam et al., 2020). Thus, we detected NADPH oxidase subtypes p47^phox^ and p67^phox^, and iNOS and SIRT1 in the post-ischemic brains. As shown in Figure 4, the expression levels of p47^phox^ and p67^phox^ was significantly up-regulated, indicating that activation of NADPH oxidases in the ischemic brains. However, the expression level of iNOS had a trend of increase in the MCAO ischemia-reperfused group but it was not statistically different from the sham control group. The increased expression of p67^phox^ was significantly inhibited by DSS treatment (*p* < 0.05). The expression of p47^phox^ and iNOS had no statistical difference between the MCAO plus vehicle group and MCAO plus DSS treatment. Meanwhile, the expression level of SIRT1 was down-regulated in the post-ischemic brains which was reserved by the DSS treatment (*p* < 0.05). Consistently, immunofluorescent staining showed that the expression of SIRT1 was increased in the cortex and striatum of the post-ischemic brains after receiving the DSS treatment (Figure 5). These results suggest that the antioxidant effects of DSS ethanol extract could be attributed to inhibiting NADPH oxidase and activate SIRT1 signaling in post-ischemic brains.

### SIRT1 Inhibitor EX527 Ablated Neuroprotective Effects of DSS Ethanol Extract Against Cerebral Ischemia-Reperfusion Injury

**FIGURE 6:**
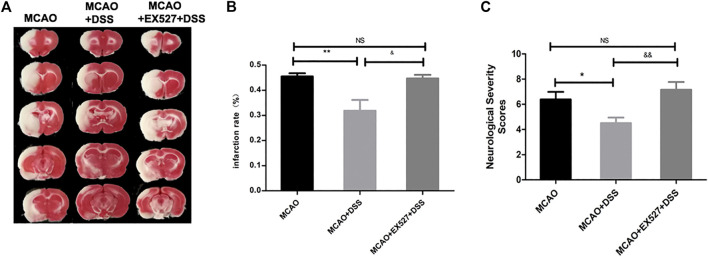
SIRT1 inhibitor EX527 ablates the neuroprotective effects of DSS. Rats were subjected to 2 h of MCAO cerebral ischemia plus 22 h of reperfusion. **(A)** Representative TTC staining for infarct sizes. **(B)** Statistical analysis of infarct sizes as measured by using Image J software. **p < 0.001, vs. MCAO + DSS group, &*p* < 0.5, vs. MCAO + EX527 + DSS group. **(C)** Statistical analysis of neurological deficit scores (mNSS), *p < 0.05, vs. MCAO + DSS group, &&*p* < 0.01, vs. MCAO + EX527 + DSS group.

We further explored whether the therapeutic effect of DSS is SIRT1-dependent. We injected SIRT1 specific inhibitor EX527 at 5 mg/kg into rat brains intraperitoneally prior to MCAO operation. DSS treatment reduced infarct size and improved neurological functions whose effects were abolished by EX527 (Figures 6). Thus, SIRT1 signaling could be one of the therapeutic targets of DSS against cerebral ischemia-reperfusion injury.

### Qualitative and Quantitative Analysis of DSS Ethanol Extract

**FIGURE 7:**
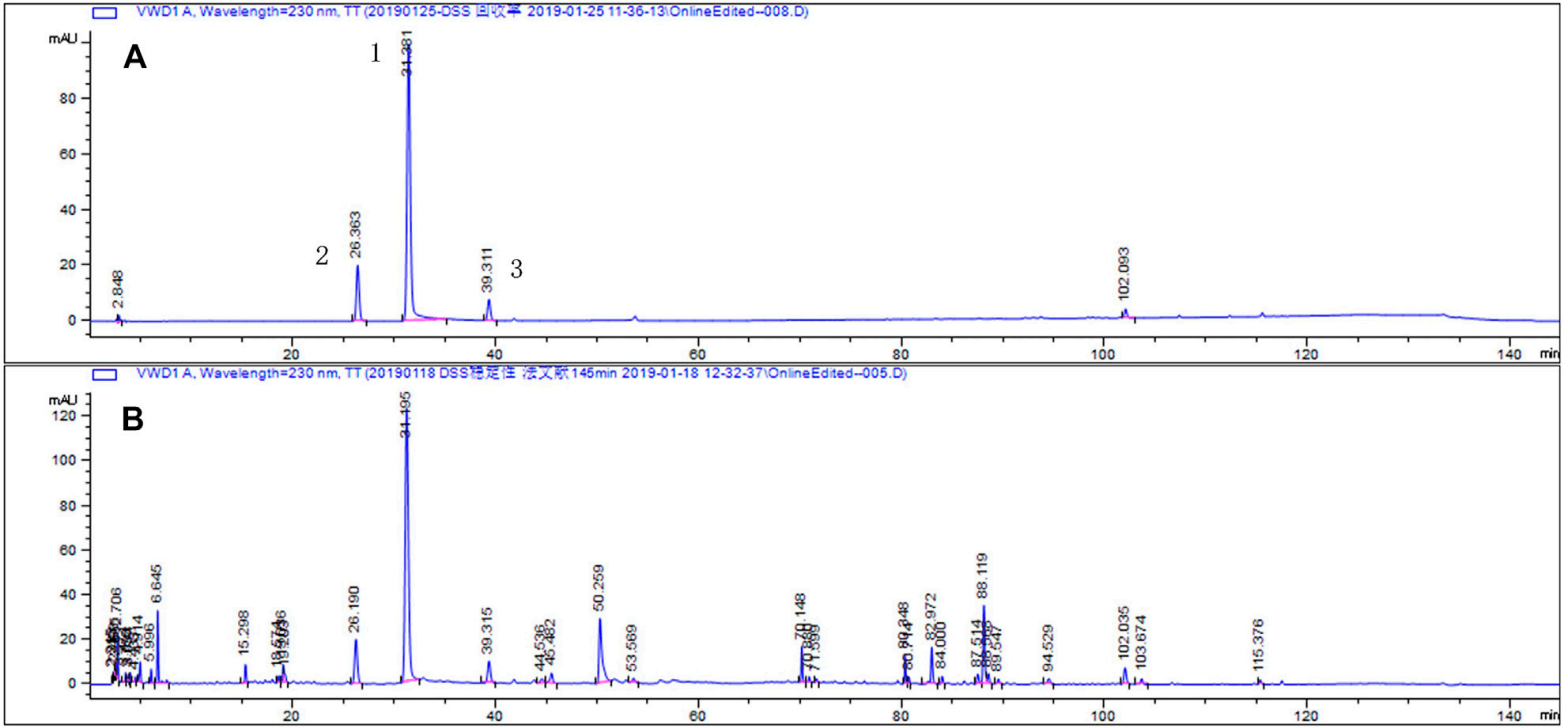
Representative chromatograms of standard compounds **(A)** and DSS ethanol extract **(B)** analyzed by HPLC-UV. 1) paeoniflorin; 2) alibiflorin; 3) feuric acid.

For the quality control of DSS/E, we identified three ingredients as the standard for HPLC analysis, including paeoniflorin, alibiflorin, and ferulic acid. The chromatographic condition was optimized and a well-separated fingerprint was obtained (Figure 7). The linearity, precision, stability, and accuracy were measured in the HPLC system (Table 1). The linarites of the standard curves for paeoniflorin, alibiflorin, and ferulic acid were y = 6.903x − 98.718 with correlation coefficients (r) 1, y = 6.903x − 17.161 with correlation coefficients 0.9994, y = 17.906x + 9.819 with correlation coefficients 1, respectively. The precisions of paeoniflorin, alibiflorin, and ferulic acid were assayed by intra-day variations (RSD) at one concentration with six replicates, which were 1.8, 1.8, and 1.6%, respectively. The stability was assessed by the RSD values of peak areas which had 1.0, 0.1, and 2.3% for paeoniflorin, alibiflorin, and ferulic acid respectively. The accuracy of the analytical method was confirmed with the overall recovery of 99.5–108.5%. These results suggest that the HPLC-UV method has good sensitivity, accuracy, and stability. With the validated HPLC-UV method, the concentrations of paeoniflorin, alibiflorin, and feuric acid were identified to be 39.7412, 5.3411, and 0.8221 μg/mg, respectively, in DSS ethanol extract.

**TABLE 1:** The linearity, precision, stability results of standard compounds paeoniflorin, alibiflorin, and ferulic acid.

Standard compounds	Regression equation (*R* ^2^) of the linearity	RSD indicator of the precision assay (%)	RSD indicator of the stability assay (%)
Paeoniflorin	Υ = 6.903X − 98.718 (1)	1.8	1.0
Alibiflorin	Υ = 6.903X − 17.161 (0.994)	1.8	0.1
Ferulic acid	Υ = 17.906X + 9.819 (1)	1.6	2.3

Correlation coefficients, *R*.^2^.

## Discussion

In the present study, we investigated the efficacies of aqueous and ethanol extract of Danggui-Shaoyao-San (DSS) against cerebral ischemia-reperfusion injury. Ethanol extract of DSS, instead of aqueous extract, significantly reduced infarct sizes and improved neurological deficit mNSS scores in the transient MCAO ischemia rats. The DSS ethanol extract inhibited the expression of NADPH oxidase subunit p67^phox^ and up-regulated SIRT1, decreased the productions of superoxide and peroxynitrite, attenuated infarct sizes and improved neurological functions in the transient MCAO ischemic rats. Those results indicate that ethanol extract of DSS has much better neuroprotective effects than the aqueous extract. The results could be used for the application of DSS in the TCM treatment for ischemic stroke.

DSS was firstly documented to be prepared with “wine” to enhance its therapeutic effects in *Essentials from the Golden Cabinet*, a classic TCM textbook written in the Eastern Han Dynasty by Master Zhongjing Zhang. A previous study reported that the organic solvent extract of DSS had higher concentrations of paeoniflorin and alibiflorin than the aqueous extract (Liu et al., 2010). Paeoniflorin has antioxidant and anti-inflammation activities and neuroprotective effects against cerebral ischemia-reperfusion injury (Tang et al., 2010; Guo et al., 2012; Zhang et al., 2015; Zhang Y. et al., 2017). Paeoniflorin increased blood supply to the ischemic hemisphere in an experimental focal cerebral ischemia-reperfusion animal model (Rao et al., 2014). Albiflorin has the capacity to pass through the BBB and protect the BBB integrity in cerebral ischemia-reperfusion injury (Li et al., 2015). Ferulic acid exerts antioxidant properties and has neuroprotective effects against cerebral ischemia/reperfusion-induced injury (Cheng et al., 2008; Cheng et al., 2016; Ren et al., 2017; Cheng et al., 2019). Thus, we used paeoniflorin, alibiflorin, and feuric acid as marker compounds for quality control whose concentrations were 39.7412, 5.3411, and 0.8221 μg/mg in DSS ethanol extract respectively.

ROS and RNS play important roles in the pathological process of cerebral ischemic-reperfusion injury (Chen et al., 2013; Chen et al., 2016; Chen et al., 2018). NADPH oxidase is a major pro-oxidant enzyme for ${\text{O}}_{2}^{-}$ generation whereas iNOS activation produces high concentration of NO. Our previous studies indicate that ischemia-reperfusion significantly up-regulated NADPH oxidase subunits p47^phox^ and p67^phox^, and iNOS and increased the production of ${\text{O}}_{2}^{-}$ and NO, subsequently inducing the production of ONOO^−^ and aggravating cerebral ischemia-reperfusion injury (Chen et al., 2015; Chen et al., 2020). Peroxynitrite has much higher toxicity and penetrating capacity across the lipid membrane than ${\text{O}}_{2}^{-}$ (Moro et al., 2005; Pacher et al., 2007). The levels of ONOO^−^ and its footprint marker 3-NT were confirmed in the cerebrospinal fluid (CSF) and plasma of stroke patients (Nanetti et al., 2007; Isobe et al., 2009). The increased ONOO^−^ production, mediates DNA damage, protein nitration and lipid peroxidation, activates matrix metalloproteinases (MMPs), degrades tight junction proteins, and aggravates the BBB disruption in ischemic brain injury (Salgo et al., 1995; Virag et al., 2003; Kuhn et al., 2004; Tajes et al., 2013; Ding et al., 2014). Thus, we used HEt and HKSOX1 to directly visualize and detected 3-NT expression in the ischemic brain tissues after the rats were exposed to 2 h of MCAO ischemia plus 22 h of reperfusion. The levels of ${\text{O}}_{2}^{-}$ and 3-NT were increased in the ischemic brains which were reduced by treatment of DSS. The expression levels of p47^phox^ and p67^phox^ were significantly increased in the ischemia-reperfused brains. The expression of iNOS had a trend of increase but without statistical differences. Treatment of DSS significantly down-regulated the expression of p67^phox^ but has no effect on the expression of p47^phox^ and iNOS statistically. Those results suggest that DSS could inhibit the production of ${\text{O}}_{2}^{-}$ and ONOO^−^ through inhibiting NADPH oxidases in the MCAO ischemic brains.

Notably, DSS ethanol extract up-regulated the expression of Silent information regulator 1 (SIRT1) in ischemic brains whose effect was abolished by EX527, a SIRT1 inhibitor. SIRT1 is a NAD^+^ dependent histone deacetylase. SIRT1 plays an essential roles in multiple cellular events including cellular stress resistance (Brunet et al., 2004; Han et al., 2017), energy metabolism (Purushotham et al., 2009; Cao et al., 2016), oxidation stress (Singh et al., 2017; Rada et al., 2018), inflammation (Yang et al., 2015), and apoptosis (Zhang M. et al., 2017; Chen et al., 2019). SIRT1 has antioxidant activity in vascular endothelial cells by modulating multiple molecular targets including FOXOs, NF-κB, NOX, SOD, and eNOS, etc. (Zhang W. et al., 2017). For example, SIRT1 inhibits NADPH oxidase activation and protects endothelial function (Zarzuelo et al., 2013). SIRT1 knockout mice had larger infarct sizes than wild-type mice after exposed to MCAO cerebral ischemia (Hernandez-Jimenez et al., 2013; Liu et al., 2013). Treatment of resveratrol, a SIRT1 activator, decreased infarct size, lessened brain edema, attenuated the BBB disruption, and improved neurological functional outcomes (Huang et al., 2001; Gao et al., 2006; Tsai et al., 2007; Cheng et al., 2009; Yousuf et al., 2009) whereas SIRT1 inhibitors aggravated ischemic brain injury (Hernandez-Jimenez et al., 2013). Thus, the antioxidant property of SIRT1 might contribute to the neuroprotective effects of DSS against cerebral ischemia-reperfusion injury. With multiple active gradients in the DSS formula, it is of interesting to explore the active compounds with the properties of regulating SIRT1 signaling. A recent study revealed that paeoniflorin attenuated ox-LDL-induced apoptosis and inhibited adhesion molecule expression via upregulating SIRT1 in endothelial cells (Wang et al., 2019). Of note, DSS has multiple constitutes (Fu et al., 2016) with complex network regulating mechanisms in ischemic brain injury, the exact molecular targets and mechanisms remain to be further elucidated.

In conclusion, DSS ethanol extract could protect against cerebral ischemic-reperfusion injury via attenuating oxidative/nitrosative stress and inhibiting neuronal apoptosis via inhibiting NADPH oxidases and activating SIRT1 signaling.

## Data Availability

The raw data supporting the conclusions of this article will be made available by the authors, without undue reservation.
