# Supplementary material for: Danggui-Shaoyao-San (DSS) Ameliorates Cerebral Ischemia-Reperfusion Injury via Activating SIRT1 Signaling and Inhibiting NADPH Oxidases
Source: Front Pharmacol. 2021 Apr 15;12:653795. doi: 10.3389/fphar.2021.653795 (PMC8082392; doi:10.3389/fphar.2021.653795)
Supplement: Supplementary file 1 [file table1.docx]

Supplement 1. HPLC gradient elution conditions

| Time（min） | Mobile phase | | Flow (ml/min） |
| --- | --- | --- | --- |
|  | Solvent A(acetonitrile) | Solvent B（0.05% phosphoric acid） |  |
| 0 | 95% | 5% | 1 |
| 0~15 | 88% | 12% | 1 |
| 15~25 | 88% | 12% | 1 |
| 25~60 | 75% | 25% | 1 |
| 60~80 | 45% | 55% | 1 |
| 80~100 | 30% | 70% | 1 |
| 100~120 | 5% | 95% | 1 |
| 120~130 | 5% | 95% | 1 |
| 130~138 | 95% | 5% | 1 |
| 138~145 | 95% | 5% | 1 |
